# Modelling disease course in amyotrophic lateral Sclerosis: pseudo-longitudinal insights from cross-sectional health-related quality of life data

**DOI:** 10.1186/s12955-020-01372-6

**Published:** 2020-05-01

**Authors:** Tino Prell, Nayana Gaur, Robert Steinbach, Otto W. Witte, Julian Grosskreutz

**Affiliations:** 1 Hans Berger Department of Neurology, Jena University Hospital, Am Klinikum 1, 07747 Jena, Germany; 2 Center for Healthy Ageing, Jena University Hospital, Jena, Germany

**Keywords:** Disease aggressiveness, Health-related quality of life, Longitudinal modelling

## Abstract

**Background:**

Amyotrophic Lateral Sclerosis (ALS) is a rapidly progressive neurodegenerative disorder with limited robust disease-modifying therapies presently available. While several treatments are aimed at improving health-related quality of life (HRQoL), longitudinal data on how QoL changes across the disease course are rare.

**Objectives:**

To explore longitudinal changes in emotional well-being and HRQoL in ALS.

**Methods:**

Of the 161 subjects initially recruited, 39 received 2 subsequent follow-up assessments at 6 and 12 months after baseline. The ALS Functional Rating Scale-Revised (ALSFRS-R) was used to assess physical impairment. HRQoL was assessed using the ALS Assessment Questionnaire (ALSAQ-40). The D50 disease progression model was applied to explore longitudinal changes in HRQoL.

**Results:**

Patients were primarily in the early semi-stable and early progressive model-derived disease phases. Non-linear correlation analyses showed that the ALSAQ-40 summary index and emotional well-being subdomain behaved differently across disease phases, indicating that the response shift occurs early in disease. Both the ALSFRS-R and ALSAQ-40 significantly declined at 6- and 12-monthly follow-ups.

**Conclusion:**

ALSAQ-40 summary index and emotional well-being change comparably over both actual time and model-derived phases, indicating that the D50 model enables pseudo-longitudinal interpretations of cross-sectional data in ALS.

## Introduction

Amyotrophic lateral sclerosis (ALS) is a fatal and relentless neurodegenerative disorder that is characterized by motor neuron degeneration and several non-motor symptoms. Patients suffer progressive wasting of the limb, bulbar and respiratory muscles and typically succumb to respiratory failure a few years after symptom onset [1]. No robust disease-modifying therapies are presently available. Treatments are therefore aimed at improving quality of life (QoL). While various factors have been reported to impact QoL and health-related QoL (HRQoL) in ALS [2, 3], little is known about how HRQoL and its sub-domains behave across the disease course. Given the aforementioned limited longevity of patients and high drop-out rates in prospective studies, longitudinal trials are rare. The D50 model was developed to help address these constraints [4]; it uses ALS Functional Rating Scale-Revised (ALSFRS-R) scores to describe individual disease trajectories. In the present study, we **i)** applied the D50 model to a large cross-sectional HRQoL dataset and **ii)** compared these results with a sub-sample of longitudinal HRQoL data.

## Methods

### Subject recruitment and assessment

Patients with ALS with a diagnosis of definite, probable, laboratory-supported probable, or possible ALS (as determined by the revised El-Escorial criteria [5]) without dementia were consecutively recruited from the Department of Neurology, Jena University Hospital between May 2013 and December 2018. All diagnoses were assigned by a team of expert attending neurologists. Written informed consent was obtained from all participants and the study was approved by the local Ethics committee of the Jena University Hospital. Of the 161 subjects initially recruited, 39 received two subsequent follow-up assessments at 6 and 12 months after baseline.

The ALSFRS-R was used to assess physical impairment [6]. HRQoL was assessed using the ALS Assessment Questionnaire (ALSAQ-40) [7]; this spans the domains of mobility, activities of daily living (ADL), eating, communication, and emotional well-being. The ALSAQ-40 summary index (SI) was calculated as a measure of overall HRQoL, with higher values indicating poorer HRQoL.

### The D50 model: overview and application

**Fig. 1:**
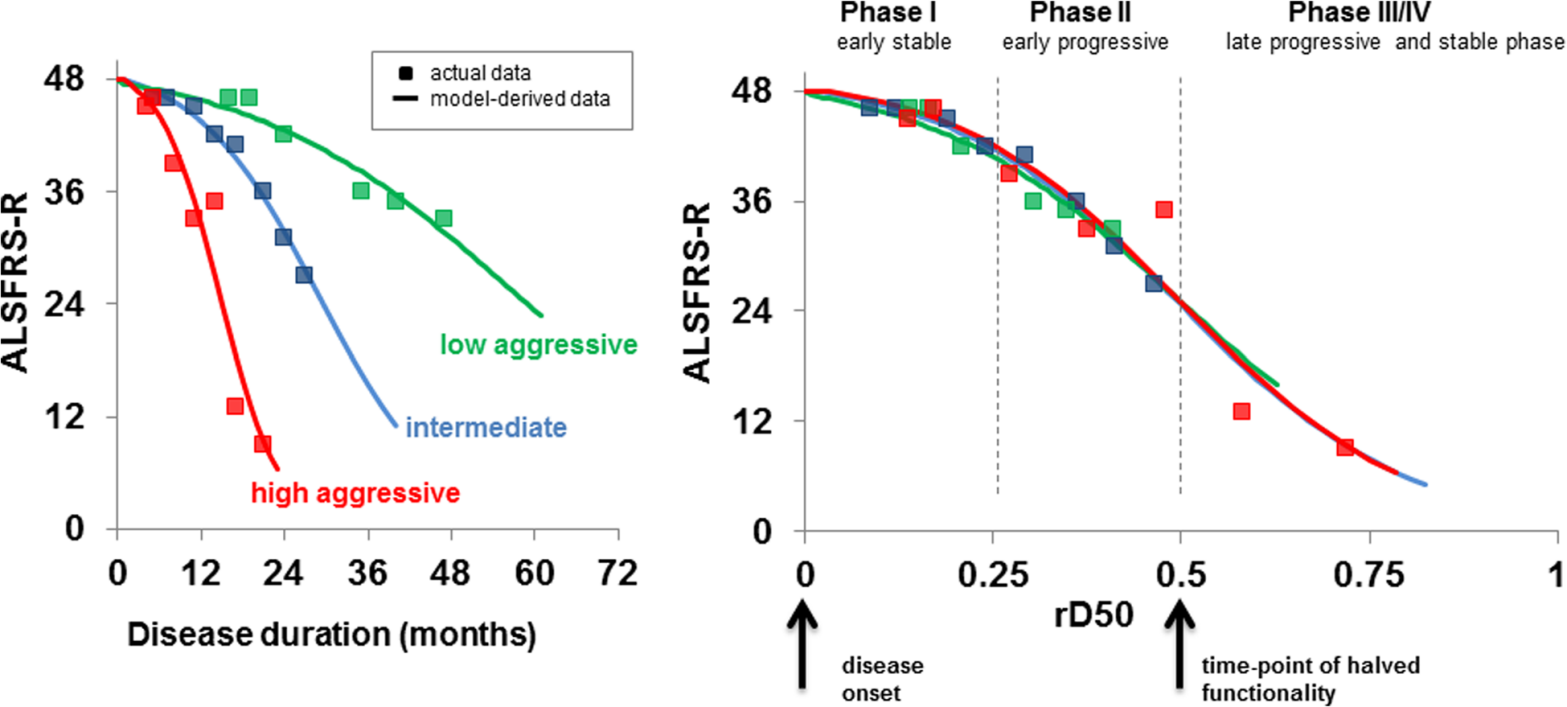
The D50 model and rD50-derived disease phases. The model yields three key descriptive parameters: D50 as the time taken in months for ALSFRS-score to drop to 24 points, dx as the time constant of ALSFRS-R decay, and the rD50, which describes individual disease course covered in reference to D50. **a** D50 and dx are calculated from actual ALSFRS-R scores for 3 representative patients with distinct disease profiles; high, moderate, and low disease aggressiveness. **b** Normalization with rD50 allows for comparability between patients with vastly different disease time scales and shows that patients proceed through similar phases of functional decline independent of individual disease aggressiveness

The D50 model describes the disease course of individual patients with ALS as a sigmoidal state transition from full health to complete functional loss. The curve is calculated by iterative fitting of regularly collected ALSFRS-R scores that are available for a given patient. The model takes into account that progression in ALS is non-linear and highly heterogeneous [8]. As a result, the D50 model provides measures of overall disease aggressiveness, local disease activity, and disease phases [4, 9]. D50 is the estimated time taken in months for the ALSFRS-R to drop to 24 and can therefore summatively describe individual disease aggressiveness independent of the assessment time-point. Normalizing individual D50 values to 0.5 yields the parameter relative D50 (rD50). The rD50 is an open-ended reference point where 0 signifies disease onset and 0.5 indicates the time-point of halved functionality. By virtue of its normalized framework, rD50 allows comparability between patients with vastly different disease courses. Based on individual rD50 values, patients can be categorized into mathematically-derived disease phases: an early semi-stable Phase I (0 ≤ rD50 < 0.25), an early progressive Phase II (0.25 ≤ rD50 < 0.5), and late progressive and late stable Phases III/IV (≥ 0.5) (Fig. 1) [10, 11]. Crucially, rD50 is not to be conflated with or used interchangeably with disease duration in months.

### Statistics

SPSS (version 25.0; IBM Corporation, Armonk, NY, USA) was used for all statistical analyses. Correlations between rD50 and **a)** the ALSAQ40 SI and **b)** the emotional well-being sub-domain were visualized using locally weighted scatterplot smoothing (LOESS). A repeated-measures ANOVA (with a Greenhouse-Geisser correction to correct for violations of sphericity) was used in combination with the Bonferroni *post-hoc* test to analyze longitudinal data. A repeated measures ANOVA was used as this was an exploratory study and because the test is relatively robust against sphericity and normality violations. Statistical significance was set at *p* < 0.05.

## Results

**Fig. 2:**
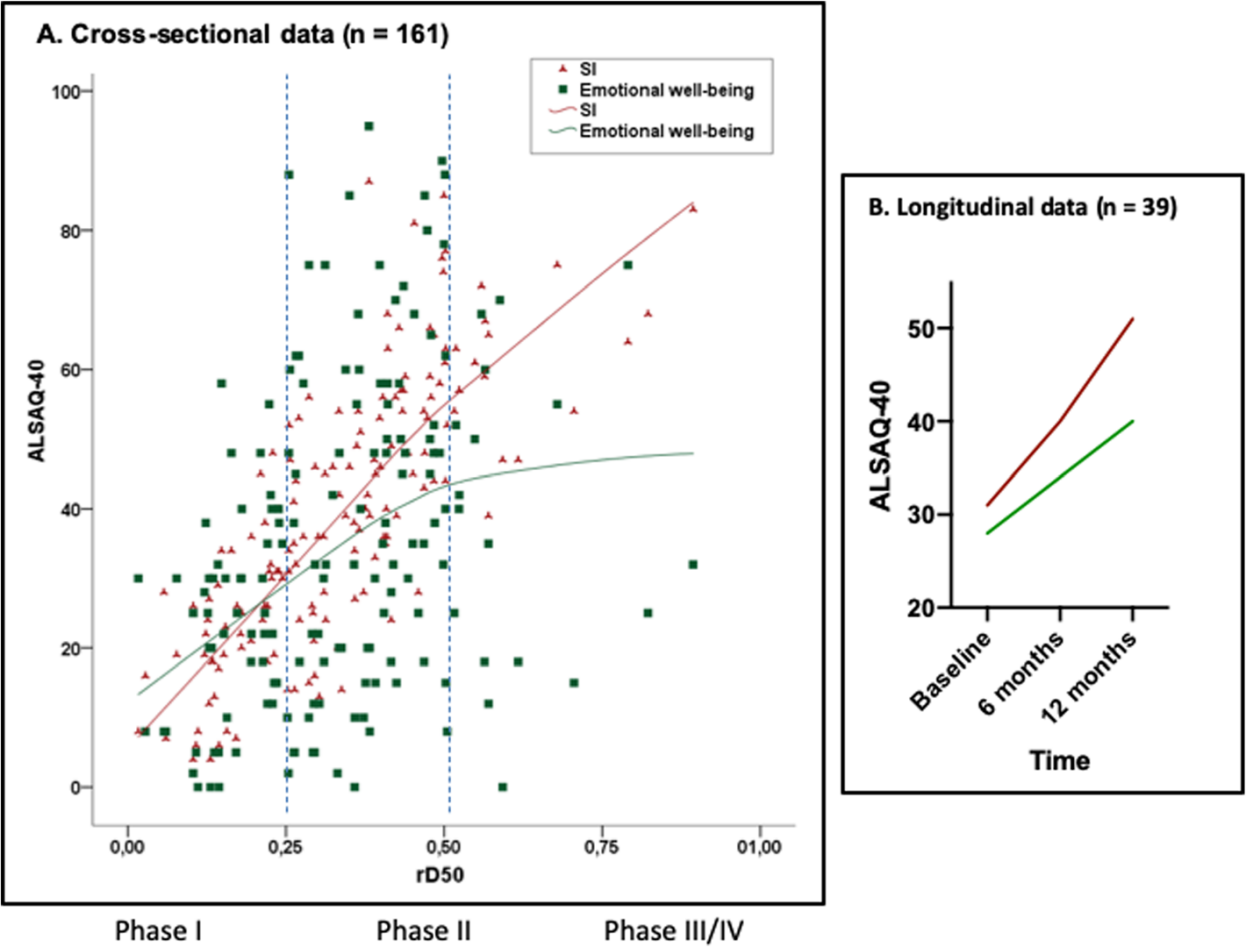
ALSAQ-40 and clinical parameters. a The non-parametric regression Loess (Epanechnikov) was used to visualize the behavior of the ALSAQ-40 SI and emotional well-being across the rD50-derived disease phases in the cross-sectional data. **b** Mean values of ALSAQ-40 SI and emotional well-being at baseline and first (6 months) and second (12 months) follow ups

Clinical and demographic data are detailed in Table 1. Based on rD50 values, the majority of patients were in disease Phases I and II (Table 1). Figure 2a shows how LOESS can help visualize the behavior of the ALSAQ-40 SI and the emotional well-being sub-domain across rD50-derived disease phases. A comparable increase in both the ALSAQ-40 SI and emotional well-being sub-domain values can be seen in Phase I; these translate to a concomitant decrease in HRQoL. The ALSAQ-40 SI value continues to increase in Phase II, while the slope of the emotional well-being sub-domain is deflected. In Phases III/IV, a clear divergence of slopes is evident for the ALSAQ-40 SI and the emotional well-being sub-domain, with the latter showing a particularly heterogeneous distribution between patients. The mobility and ADL sub-domains followed a similar trajectory to that of the ALSAQ-40 SI (data not shown).

**Table 1:** Participant demographics and clinical data

Continuous Variables	Mean	SD
Age	61.8	11.5
Disease duration in months	23	20
*Disease severity*		
• D50	36.0	24.0
• ALSFRS-R total	34.8	8.6
• ALSFRS-R bulbar (1–3)	8.6	3.4
• ALSFRS-R cervical (4–6)	6.8	3.7
• ALSFRS-R lumbar (7–9)	6.5	3.7
• ALSFRS-R thoracic (10–12)	9.8	2.5
*Health-related Quality of Life*		
• ALSAQ-40		
• ALSAQ-40 Mobility	40.3	30.1
• ALSAQ-40 Activities of daily living	44.0	31.6
• ALSAQ-40 Eating	24.4	29.6
• ALSAQ-40 Communication	43.2	36.2
• ALSAQ-40 Emotional well-being	34.6	23.1
Categorical Variables	**n**	**%**
Male	95	59
Female	66	41
Limb onset	104	64.6
Bulbar onset	57	35.4
*rD50-derived Disease Phase*		
I	54	33.5
II	89	55.3
III/IV	18	11.2

Thirty-nine patients (20 females; 12 bulbar- and 27 limb-onset) received 2 subsequent follow-up assessments at 6 and 12 months after baseline. The mean ALSFRS-R reduction for these 39 patients across these time points was as follows: 39 ± 5.3 (baseline) to 34 ± 6.7 (6 months) and finally to 28 ± 9.5 (12 months). The repeated-measures ANOVA revealed that the mean ALSAQ-40 SI significantly increased across this period (*F* (1.53, 54.4) = 28.6; *p* < 0.001; partial η^2^ = 0.44; mean ALSAQ-40 SI at baseline 31 ± 15, 6 months 40 ± 17, 12 months 51 ± 20) (Fig. 2b). The emotional well-being sub-domain scores increased from baseline (28 ± 19) to both the first (34 ± 29, *p* = 0.06) and second follow-ups (40 ± 24; *F* (1.4, 50.6) = 8.9; *p* = 0.002; partial η^2^ = 0.2) (Fig. 2b).

## Discussion

The present longitudinal study confirms the association between physical impairment and HRQoL in ALS and the results reported here are in line with those reported by similar studies [12–14]. Whether physical impairment in ALS follows the same trajectory as QoL/HRQoL primarily depends on the instrument used to assess well-being. Therefore, the findings observed here are expected, given that we used the ALSAQ-40, which is heavily weighted towards physical function. Previous studies in the field have reported little change in QoL, despite a profound worsening of physical function [15–17]. This phenomenon is commonly observed in several chronic disorders and is referred to as a “*response shift*” [18]. The response shift potentially manifests as a result of patients recalibrating their views on life, their condition, and their internal standards for QoL. In the context of ALS, this translates to patients experiencing a change in the factors that they believe contribute most significantly to their QoL as their physical strength wanes [3, 19]. Here, we show that the ALSAQ-40 SI, and the mobility and emotional well-being sub-domains behave asymmetrically during functional disease phases and across the absolute disease course in months. We posit that this underlies the response shift in ALS.

This leads to the second crucial finding of the present study. We show here that the D50 model can closely approximate longitudinal results from a cross-sectional dataset i.e. results obtained using pseudo-longitudinal modelling parallel those observed at “true” longitudinal follow-ups. The ALSAQ-40 SI and emotional well-being domain followed the same trajectory in a sub-cohort that was followed up at 6 and 12 months after baseline. We believe these results underscore the advantages of the D50 model and the utility of a relative index like rD50. This approach affords the possibility of studying outcomes which would ordinarily only be possible with longitudinal data. For the study of an aggressive disease like ALS, the usefulness of such an approach cannot be overstated. The model can be easily applied to heterogeneous ALS cohorts to interpret how disease aggressiveness and phases affect a range of variables. The present study is not without its limitations. Although our cohort size was comparable to that of other studies [14, 17], few patients were available for follow-up, thus limiting statistical power. We recommend that future confirmatory studies perform appropriate power analyses and account for the effects of additional confounding variables like genetic status and the presence of comorbidities. Furthermore, the majority of the patients were in disease Phases I and II, thus making a conclusive determination of how the D50 model performs in later stages of the disease difficult; indeed this is when the relationship between disease severity and QoL is harder to fully predict and may lie at extremes of a spectrum.

## Conclusion

In a first exploratory study, we have shown that the ALSAQ-40 summary index and emotional well-being show comparable longitudinal and pseudo-longitudinal changes, indicating the D50 model’s utility for studying phenomena across the ALS disease course. Future prospective studies following larger cohorts with slowly progressing patients over a longer period could corroborate and complement our results.

## Data Availability

Data from this study will be shared with qualified investigators upon reasonable request for scientific purposes.

## Abbreviations

ADL: Activities of Daily Living
ALS: Amyotrophic Lateral Sclerosis
ALSAQ-40: Amyotrophic Lateral Sclerosis Assessment Questionnaire
ALSFRS-R: Amyotrophic Lateral Sclerosis Functional Rating Scale Revised
ECAS: Edinburgh Cognitive and Behavioral Amyotrophic Lateral Sclerosis Screen
HRQoL: Health-Related Quality of Life
LOESS: Locally Weighted Scatterplot Smoothing
QoL: Quality of Life
rD50: Relative D50
SI: Summary Index
